# In vivo and *ex vivo* sonographic evaluation of tumor margins during micrographic‐controlled surgery: a promising new tool in dermato‐oncology?

**DOI:** 10.1111/ddg.15827

**Published:** 2025-07-02

**Authors:** Diana Crisan, Ximena Wortsman, Fernando Alfageme, Karin Scharffetter‐Kochanek, Maria Crisan, Lukas Bernhard, Evelyne Tarnowietzki, Lars Alexander Schneider, Monika‐H. Schmid‐Wendtner

**Affiliations:** ^1^ Clinic of Dermatology and Allergology University Clinic Ulm Ulm Germany; ^2^ Department of Dermatology School of Medicine Pontificia Universidad Catolica de Chile Santiago Chile; ^3^ Department of Dermatology Faculty of Medicine Universidad de Chile Santiago Chile; ^4^ Institute for Diagnostic Imaging and Research of the Skin and Soft Tissues Santiago Chile; ^5^ Department of Dermatology and Cutaneous Surgery University of Miami Miller School of Medicine Miami USA; ^6^ Dermatology Hospital Universitario Puerta De Hierro Majadahonda Madrid Spain; ^7^ Dermatology University of Medicine and Pharmacy Iuliu Hatieganu Cluj‐Napoca Romania; ^8^ Interdisciplinary Oncological Center Munich Germany; ^9^ Department of Dermatology and Allergology Ludwig‐Maximilian University Munich Germany

**Keywords:** dermatosurgery, imaging, micrographic controlled surgery, non‐melanoma skin cancer, skin cancer, ultrasound

## Abstract

**Background and Objectives:**

The current research examined the In vivo and *ex vivo* use of high‐frequency ultrasound (HFUS) during micrographic‐controlled surgery (MCS) of non‐melanoma skin cancer (NMSC) of the head‐and‐face area, particularly the perioperative surgical margin assessment for potential reduction in surgical steps and patients’ hospitalization time.

**Patients and Methods:**

Pre‐operative in vivo and *ex vivo* sonographies of 136 NMSCs from 111 patients were evaluated retrospectively for tumor margin assessment during MCS by an experienced dermatosurgeon, while the specimens were independently assessed by a histopathologist.

**Results:**

Our *ex‐vivo* tumor margin assessment showed a specificity of >98% for squamous cell and basal cell carcinoma, with only five false‐negative and two false‐positive results, when compared to histopathology. HFUS correctly identified tumor‐free margins in 89% of investigated lesions after first resection, as well as eight incomplete excisions (6%) in which a re‐excision was performed and tumor residues were detected histologically. Overall, HFUS showed a very high accuracy in detecting both cancer‐free margins and remaining tumor residues.

**Conclusions:**

HFUS is a highly accurate method for rapidly assessing tumor margins during MCS of NMSC of highly esthetic areas, having the potential to significantly reduce surgical steps and hospitalization time in dermatosurgical patients, improving the quality of healthcare.

## INTRODUCTION

High‐frequency ultrasound (HFUS) is a modern, non‐invasive imaging method, increasingly used in dermato‐oncological surgery for visualization and characterization of skin tumors in terms of size, shape, relationship to neurovascular, cartilaginous or bony structures as well as vascularization assessment.^1^ The information provided by HFUS prior to therapy is essential for patient counselling and selection of the best therapeutic approach in accordance with oncological guidelines, while preserving the functional and aesthetic outcome in our patients.^2^, ^3^, ^4^ The most common types of nonmelanoma skin cancers (NMSCs) are basal cell carcinomas (BCCs) and squamous cell carcinomas (SCCs), with ultraviolet (UV) exposure as the main etiological factor; hence, the cephalic extremity is a common location for their development.^5^ Especially in the head‐and‐face area, the risk of incomplete primary resection is higher than on the trunk.^6^ These tumors are often surgically removed with narrow surgical margins, by Mohs surgery or micrographic controlled surgery (MCS), in order to spare healthy tissue, preserve cosmesis and functionality, and reduce the extent of surgical reconstruction.^7^ According to the literature, the risk of local recurrence for SCC and BCC is significantly lower when MCS is performed.^8^

Mohs surgery enables the intraoperative circumferential and deep tumor margin assessment, examined by staged resection and fast histopathological evaluation. It is a highly effective tool for the rapid removal of high‐risk tumors.^9^ Nonetheless, this technique usually requires multiple surgical interventions, is time‐consuming, costly, and highly dependent on the operator's expertise, which limits its use. Slow‐Mohs surgery or MCS, often performed in German centers, refers to a staged surgical excision, where the specimens are sent to histopathological departments for processing, with the pathology results usually provided after 1–2 days. In cases of histologically proven clear margins (R0 situation) the defect is closed; in cases of tumor infiltrated margins (R1), a re‐excision is performed and the patients await the histological evaluation of the re‐resection. Depending on tumor subtype, multiple re‐resections may sometimes be necessary, prolonging patients’ hospitalization, increasing healthcare costs and the risk of perioperative complications in mostly elderly patients.^10^

Compared to dermoscopy, confocal microscopy (CM), and optical coherence tomography (OCT), HFUS has no limitation in penetration depth when evaluating skin tumors, whereas most optical techniques reach only a few millimeters into the tissue.^10^, ^11^ Pre‐operative HFUS provides real‐time three‐dimensional information on tumors, enabling clinicians to plan their excisions more precisely, accurately determining resection margins and lowering recurrence rates.^12^ In our retrospective study, we assessed the efficacy of pre‐operative in vivo and intra‐operative *ex vivo* HFUS for tumor margins identification during MCS of NMSC of the head and face region prior to histological assessment and correlated the results with the histological findings. Furthermore, we evaluated the capacity of *ex vivo* ultrasound to reduce the number of surgical steps during MCS, by predicting tumor‐free margins.

## PATIENTS AND METHODS

We conducted a retrospective chart review of 111 patients presenting to our Dermatosurgical Department (Clinic of Dermatology and Allergic Diseases, University Clinic Ulm, Germany) for MCS of NMSCs, specifically BCCs and SCCs, of the head and face area, between July 2023 and September 2024. Firstly, the tumor resection margins were drawn by the surgeon according to the clinical aspect; consequently, a pre‐operative sonographic assessment of the lesion was performed, evaluating the form, lateral and depth infiltration (mm). The lateral tumor borders as seen on ultrasound were marked and/or adjusted accordingly during the sonographic assessment just prior to surgery using a surgical skin marker.

For difficult anatomical areas such as nostrils, concha, triangular fossa, or periorbital region, we used copious amount of gel below the hockey stick probe to get high‐quality sonograms without artifacts and placed appropriate small dressings in the nostrils or auditory canal to prevent gel accumulation. For the sonographic assessment of hyperkeratotic tumors or tumors with hemorrhagic crusts, these were removed prior to the examination to avoid artifacts that might influence the sonographic evaluation, especially the correct identification of the deep margins. The tumors were consequently resected according to the lateral border marking with a minimal safety margin. The sonographic assessment was performed using a Mindray device (TE5 multitouch ultrasound system, Mindray Medical, Shenzhen) with a linear array hockey stick transducer (L16‐4 MHz) or a Canon device (Aplio A Series) with an ultrabroadband linear array hockey stick transducer (L17‐7 MHz). For frequencies between 15–18 MHz, the axial spatial resolution is known to be 100 µm/pixel and the lateral spatial resolution 200–300 µm/pixel, while the penetration depth is adjustable (0.1–60 mm). The same type of high‐frequency transducer was used, as most probes today are multifrequency.^1^, ^13^, ^14^ The hockey stick probe is particularly well‐suited for use in the facial area due to its small dimensions, making it especially practical for scanning lesions in the nasal, auricular, or periorbital regions.

For *ex vivo* assessment – similar to the in vivo approach – the excised specimen, marked with a thread, was placed on a gauze, and a copious amount of gel was applied. The covered ultrasound probe was then positioned perpendicularly on the tumor, either in vivo or *ex vivo*, and a slow sweep was performed in both axes to identify the lesion's margins. These were identified as hypoechoic areas, compared to normal surrounding tissue. In cases of sonographic suspicion of margin involvement, a localized excision was performed, both samples being sent for pathological assessment. The defect following tumor resection was covered with a dressing for 1–2 days while awaiting histopathological results.

Patients were selected according to the following inclusion criteria: suspicion or histological confirmation of BCC or SCC, localization in the head and face area, and availability of complete clinical and sonographic data. Exclusion criteria included superficial lesions, qualifying for non‐surgical therapy, or patients with incomplete chart data. The same operator with extensive experience in HFUS performed all perioperative sonographic assessments (D.C.). The histological evaluation was performed by the dermatohistological laboratory in the dermatologic Clinic of Ulm University.

All procedures followed the ethical standards of the 2013 Declaration of Helsinki, and our retrospective study (chart review) was approved by the Medical Ethics Committee of Ulm University (No. 324/24). For statistical analysis, SPSS, version 25 (IBM Corp., Armonk, NY, USA), was used. For data evaluation, descriptive statistics and frequencies were used; continuous data were presented as a means with standard deviation (SD).

## RESULTS

We identified 136 tumors from 111 patients (40 male, 71 female), with a mean age of 76 ± 12.5 years, where in vivo and *ex vivo* sonography was performed during MCS of SCC or BCC of the head and face area between July 2023 and September 2024. The tumors included 83 BCCs (61%), out of which 38 (48.7%) were high‐risk subtypes (sclerodermiform, infiltrative, micronodular, multicentric) and 53 SCCs (39%). The mean BCC thickness was 2.5 ± 1.7 mm, while for SCC it was 3.9 ± 2.7 mm.

**FIGURE 1:**
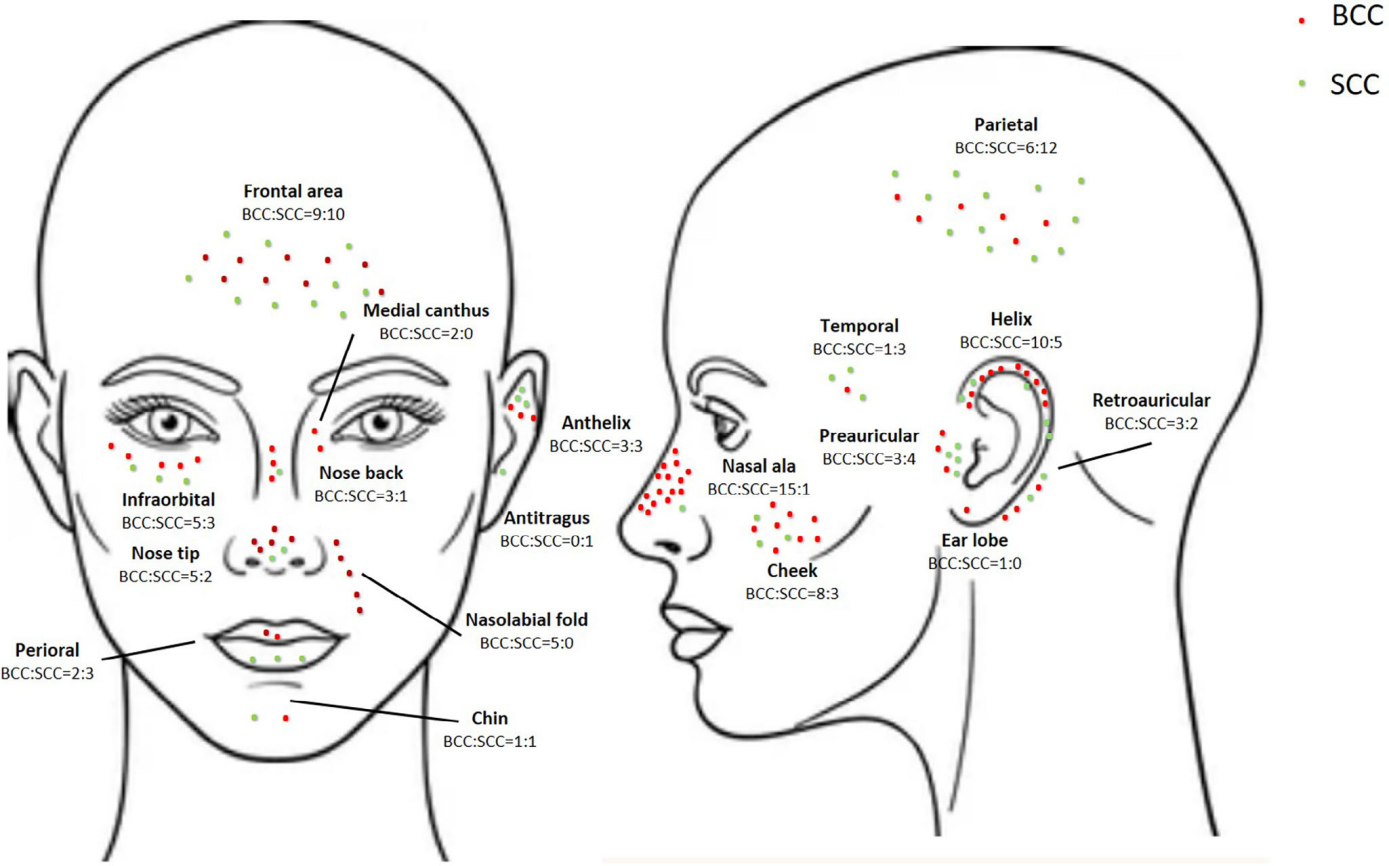
Distribution of BCCs and SCCs according to anatomical subunit in our patient cohort.

The distribution of tumors by localization and histological subtype is shown in Figure 1 and Table 1.

**TABLE 1:** Distribution of tumors according to histological subtype.

Tumor type	Tumors (n)
*Basal cell carcinoma (BCC)*	*83*
Sclerodermiform	15
Infiltrative	10
Micronodular	2
Superficial/multicentric	11
Basosquamous	2
Ulcerated	6
Solid/pigmented	37
*Squamous cell carcinoma (SCC)*	53
Well differentiated	14
Middle differentiated	6
Poorly differentiated	9
Ulcerated	4
Not classified	20

**FIGURE 2:**
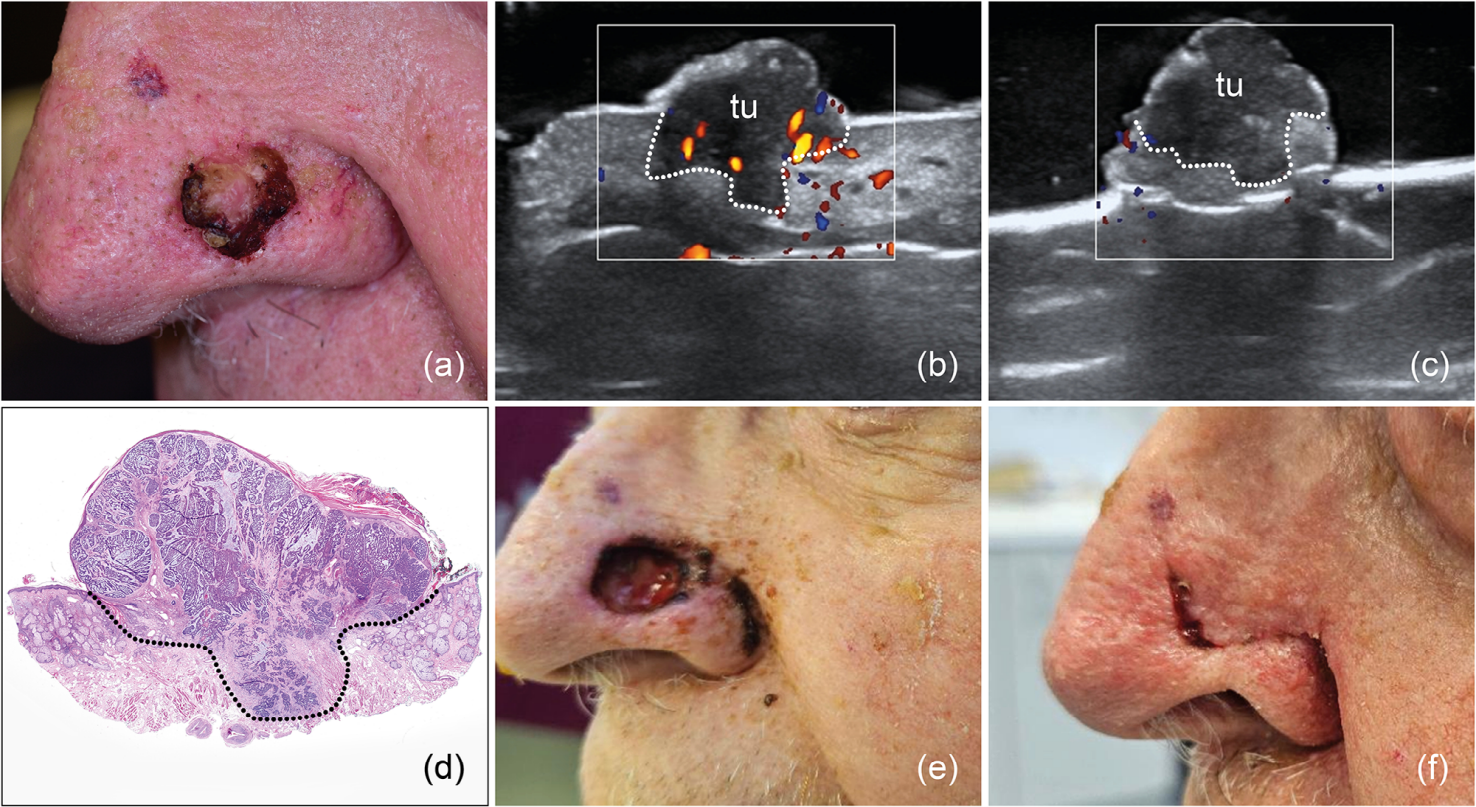
(a) Clinical aspect of a solid‐cystic BCC located at the left nasal ala; (b) In vivo sonography displaying a hypoechoic lesion with a few hyperechoic spots and basal vascularization, located in the dermis, with a tumor protrusion deep into the subcutis, marked by “tu”; (c) *ex vivo* sonography of the resected tumor, marked by “tu”, showing minimal distance to the basal excision margin and free lateral margins; (d) histological aspect of the excised specimen showing tumor‐free margins on all sides, with the specimen's appearance closely resembling the sonographic findings (hematoxylin‐eosin stain); (e) surgical defect at the nasal ala after tumor resection; (f) surgical site one week after defect reconstruction with an advancement flap.

**FIGURE 3:**
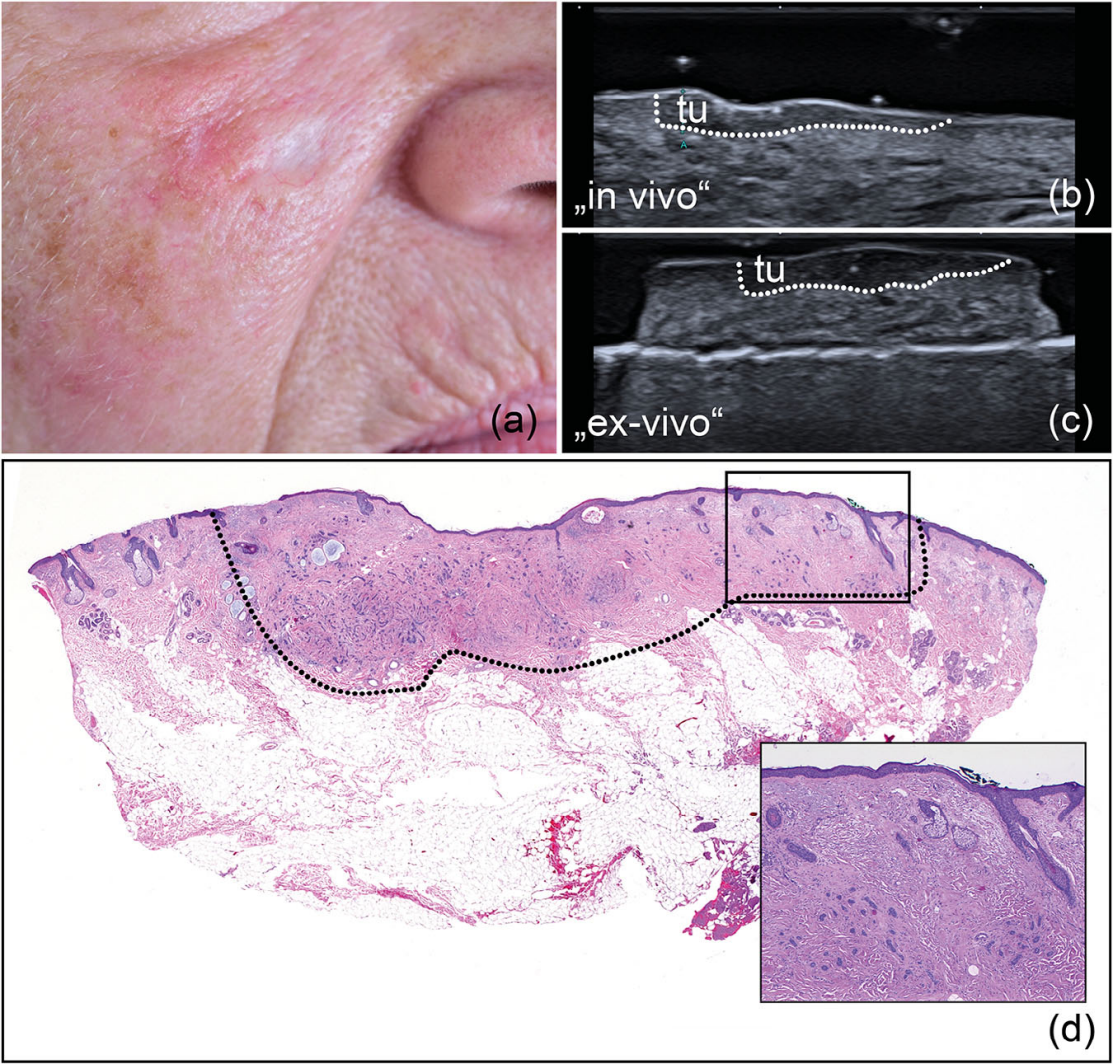
(a) Clinical aspect of a whitish, poorly defined sclerodermiform BCC at the right cheek; (b) In vivo sonography displaying a hypoechoic lesion with numerous hyperechoic spots, located in the dermis and marked by “tu”; (c) *ex vivo* sonography of the resected tumor, marked by “tu”, showing a safety margin at both the basal and lateral excision margins; (d) histological aspect of the excised specimen showing tumor‐free margins on all sides, with the specimen's appearance closely resembling the sonographic findings; inset showing histological magnification (x 40) with singular tumor nests at the lateral border of the lesion (hematoxylin‐eosin stain).

**FIGURE 4:**
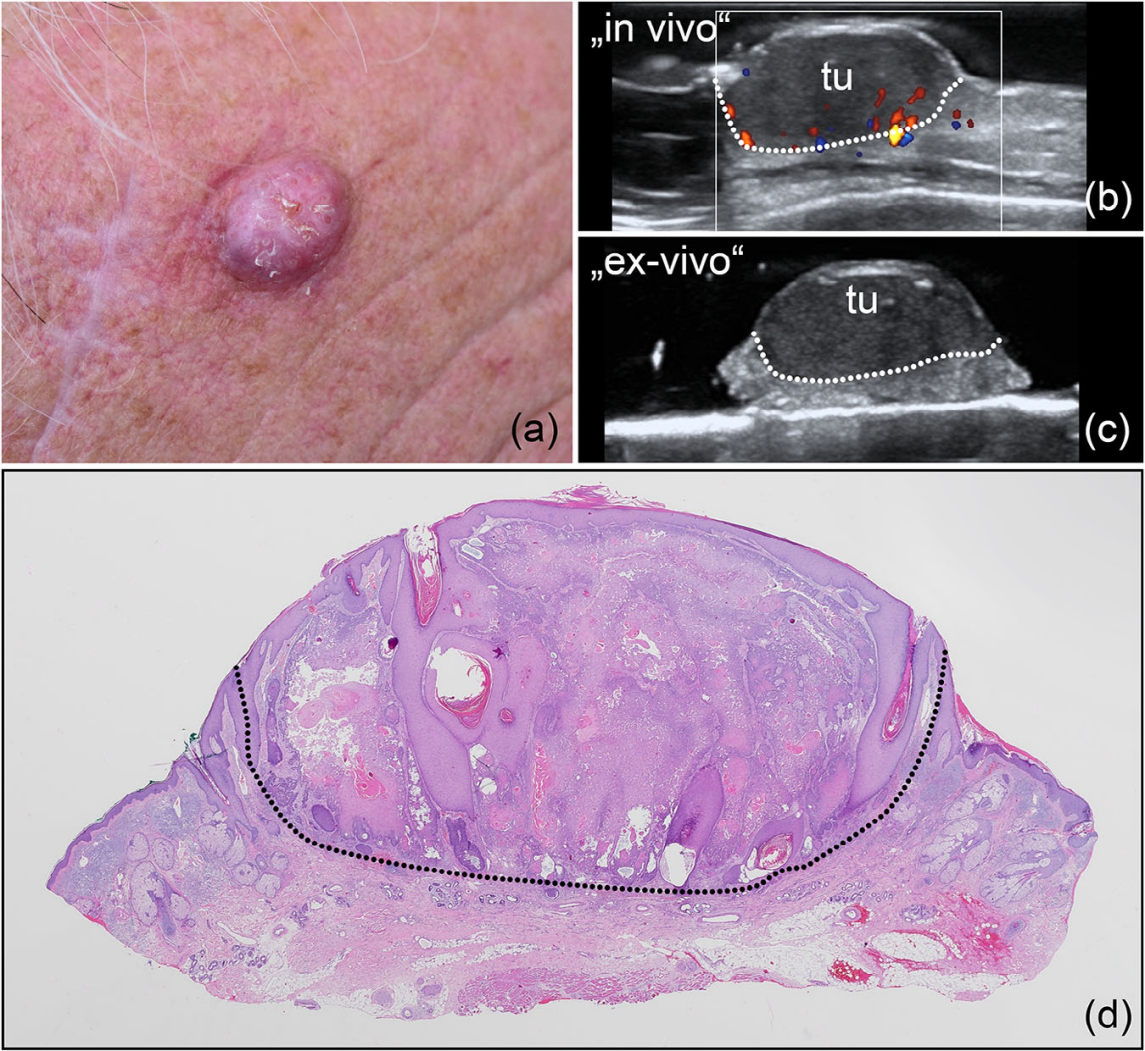
(a) Clinical aspect of an erythematous nodule located at the right frontal area, consistent with an SCC; (b) In vivo sonography displaying a hypoechoic lesion in the dermis and subcutis, marked by “tu”, with increased basal vascularization, as shown by color Doppler; (c) *ex vivo* sonography of the resected tumor, marked by “tu”, showing tumor‐free margins laterally and in depth; (d) histological aspect of the excised specimen, confirming an acantholytic‐cystic SCC, completely resected (hematoxylin‐eosin stain).

**FIGURE 5:**
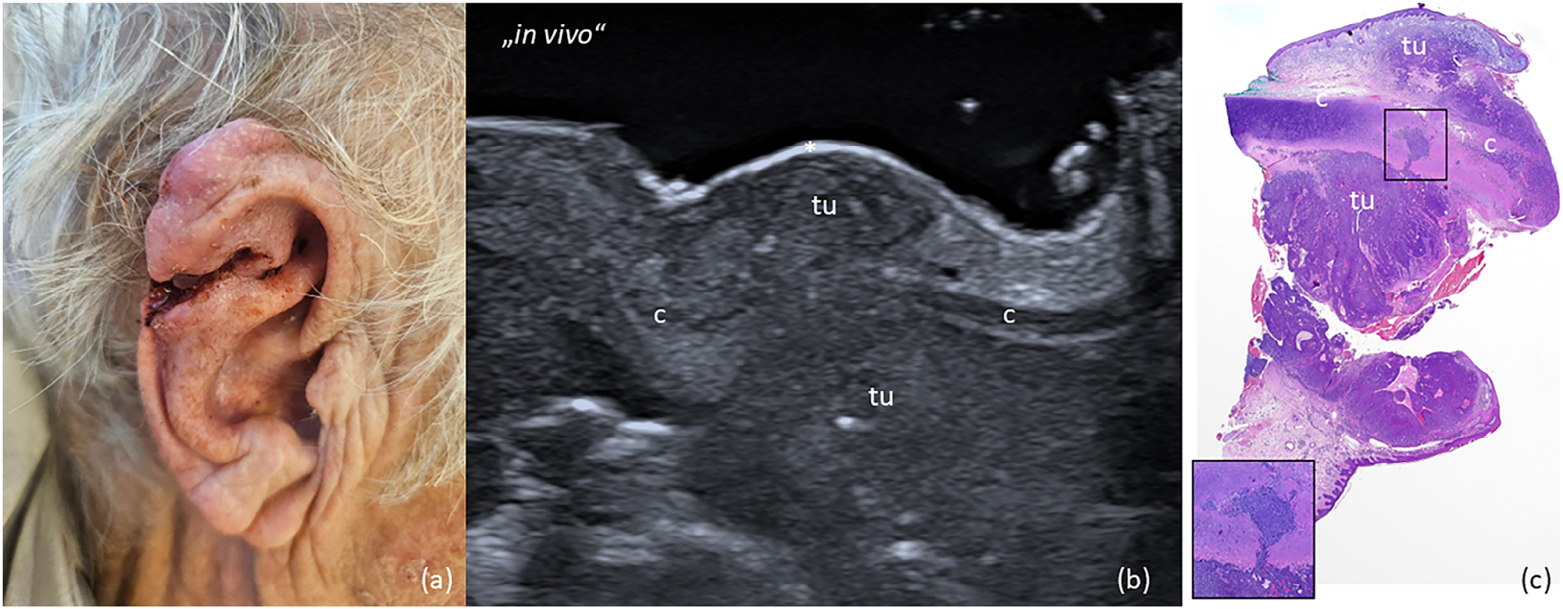
(a) Clinical aspect of an ulcerated BCC at the right auricular area; (b) In vivo sonography displaying a hypoechoic lesion, marked by “tu”, invading the cartilage, marked by “*”; (c) histological aspect of the excised specimen, confirming localized cartilage infiltration, as shown in the inset (hematoxylin‐eosin stain, original magnification x 40).

**FIGURE 6:**
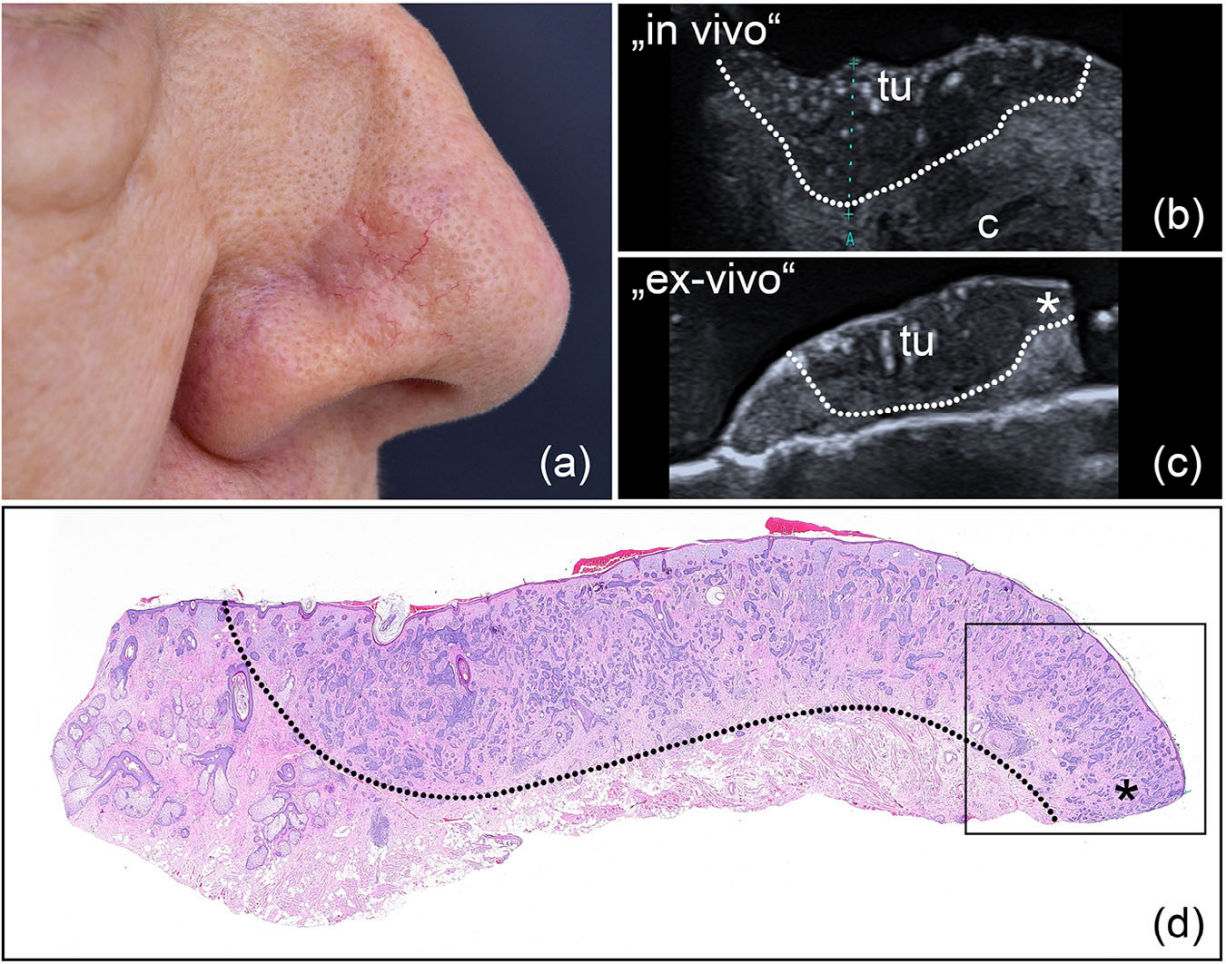
(a) Clinical aspect of a sclerodermiform BCC at the right nasal ala; (b) In vivo sonography displaying a hypoechoic lesion in the dermis and hypodermis with multiple hyperechoic spots, consistent with a high‐risk subtype, marked by “tu”; the cartilage, marked by “c”, is not infiltrated by the tumor; (c) *ex vivo* sonography of the resected tumor, marked by “tu”, showing tumor‐free margins on one side and in depth, but with suspicion of residual tumor at the other side, marked by “*”; (d) histological aspect of the excised specimen, confirming tumor invasion at one lateral margin, marked by “*” (hematoxylin‐eosin stain).

Using pre‐operative in vivo and intra‐operative *ex vivo* HFUS for margin assessment, 121 (89%) of primary tumor resections were classified as tumor‐free (R0), with histology confirming the findings (Figures 2, 3, 4). Furthermore, pre‐operative sonography correctly identified tumor infiltration of muscle/cartilage in all investigated cases of SCC and BCC (5 tumors), enabling a complete single‐step resection (Figure 5). Six BCCs, mostly high‐risk subtypes (1 sclerodermiform, 2 infiltrative, 1 multicentric, 1 micronodular, 1 solid) were sonographically assessed R1 either to the side or deep margin and re‐excised; tumor rests were histologically found in all cases (Figure 6).

One patient with a solid forehead BCC showed superficial tumor remnants laterally (TD 0,3 mm); however, he decided against re‐excision and in favor of local therapy and follow‐up. One case of micronodular BCC of the nasal ala was considered uncertain R0 on *ex vivo* ultrasound, and a localized re‐resection was performed; histology could not identify any tumor residues.

Four cases of BCCs (3 sclerodermiform – helix, parietal, 1 basosquamous – helix), were declared completely resected on *ex vivo* ultrasound and the histological assessment revealed a R1 situation. For the helical basosquamous carcinoma, histology showed singular tumor cells and perineural infiltration along the cartilage, which was missed on HFUS. Two helical sclerodermiform BCCs showed deep R1 margins on histology; therefore, re‐excisions were performed. However no tumor cells could be identified in or around the cartilage. One sclerodermiform BCC in the parietal area was considered R0 based on sonography, with histology showing a lateral R1 margin; however, re‐excision at that level was tumor‐free. The resection margins of the remaining 72 investigated BCCs were tumor‐free in both sonographic and histologic assessments.

Three SCCs were classified as R1 on *ex vivo* ultrasound and were re‐excised. Two of these SCCs (one ulcerated, one poorly differentiated) showed deep positive margins in histology, and residual tumor was found in the re‐excision specimens. For the third moderately differentiated parietal SCC, *ex vivo* ultrasound indicated the need for re‐resection; however, no residual tumor was found histologically.

One low differentiated SCC of the nose tip was considered R0 by sonography, whereas histology showed a R1 situation, requiring two re‐excisions to achieve tumor‐free margins. The resection margins of the remaining 49 excised SCCs were classified as tumor‐free on both sonographic and histologic assessment. The concordance between *ex vivo* and histological findings, as well as the sensitivity, specificity, and diagnostic accuracy of *ex vivo* HFUS, are presented in Table 2.

**TABLE 2:** Sensitivity, specificity and accuracy concordance of the sonographic and histologic findings in our patient cohort with BCCs and SCCs.

BCC	Histology R1	Histology R0	Total
Ultrasound – R1	6	1	7
Ultrasound – R0	4	72	76
Total	10	73	83
Sensitivity	60%		
Specificity	98,6%		
Accuracy	93,98%		

SCC	Histology R1	Histology R0	Total
Ultrasound – R1	2	1	3
Ultrasound – R0	1	49	50
Total	3	50	53
Sensitivity	66,6%		
Specificity	98%		
Accuracy	96,2%		

## DISCUSSION

Mohs surgery and MCS represent precise and efficient methods for achieving tumor‐free margins, particularly in high‐risk NMSCs located in esthetic areas where tissue‐sparing surgery is crucial for optimal defect reconstruction.^15^ However, during MCS, the number of surgical steps, final defect size, and outcome are unpredictable without a reliable tool for pre‐surgical tumor margin mapping.^16^

Confocal microscopy is increasingly used in clinical practice for lateral margin assessment of BCCs and lentigo maligna and is also applied *ex vivo* on freshly excised specimens for this purpose.^10^, ^16^ While CM can identify BCC nests beyond the pre‐surgical margin, this method is limited to superficial lesions due to the shallow laser penetration depth (200 µm). Therefore, it cannot assess deep margins, which are critical in many high‐risk tumors.^17^ Similarly, OCT, using infrared light, can produce cross‐sectional images of skin tumors, achieving an imaging depth of 1.5 mm, and was shown to improve sensitivity and specificity in diagnosing BCCs while also predicting tumor extension beyond clinical margins in 80–100%.^18^, ^19^

Line‐field confocal optical coherence tomography (LC‐OCT) is a new, promising method with a penetration depth of 500 µm and a resolution of 1.1–1.3 µm. It can provide a vertical image similar to a histological section, a horizontal image comparable to reflectance confocal microscopy with cellular resolution, and a three‐dimensional view of the examined lesion.^20^ LC‐OCT is increasingly and successfully being used for the early, in vivo, non‐invasive diagnosis and therapeutic follow‐up of many skin diseases including skin neoplasms.^21^

This imaging method has also been shown to be useful in the *ex vivo* setting for assessing lateral tumor borders. However, its penetration depth is limited to the mid‐dermis, making the evaluation of larger tumors and their deep borders challenging. Nonetheless, there are robust data on the use of LC‐OCT in high‐risk BCC patients undergoing Mohs surgery. When applied *ex vivo*, LC‐OCT has been shown to significantly reduce the mean number of surgical stages compared to controls by assessing lateral tumor borders.^22^

HFUS (>15 MHz) can, in contrast, provide a very reliable peri‐operative evaluation and characterization of tumors in all dimensions, without a limitation in penetration depth.^23^, ^24^, ^25^, ^26^, ^27^ It is able to accurately identify tumor margins pre‐operatively, enabling a better tumor demarcation.^2^ In our analysis, pre‐operative US for tumor margin demarcation significantly increased the number of complete resections after only one surgical step (93% of lesions). Furthermore, high‐frequency ultrasound (HFUS) correctly identified muscle or cartilage infiltration in the pre‐operative setting (2 SCC and 3 BCC cases). This enabled direct resection of the infiltrated area, reducing the procedure by at least one surgical step or even altering the treatment approach (e.g., opting for radiotherapy instead of surgery).

One limitation of HFUS is the poor differentiation between different melanocytic lesions, which is possible by LC‐OCT, for instance, since it cannot identify pigment. Nonetheless, for pigmented lesions, such as malignant melanoma (MM), HFUS can accurately identify the tumor infiltration depth and enables the performance of a locoregional staging at time of diagnosis. The preoperative assessment of tumor infiltration depth in MM using ultrasound at the time of diagnosis can help determine the need for sentinel lymph node biopsy and the appropriate safety margins.^23^

Pasquali et al. first reported on the use of a 22 MHz HFUS device (Taberna ProMedicum, Lüneburg, Germany) for *ex vivo* margin assessment. However, the investigated lesions could not exceed 13 mm in length or 8 mm in depth, as these were the maximum dimensions assessable by the equipment. Seventy‐nine out of 100 lesions were BCCs, mostly nodular; 16 were benign lesions (nevi, dermatofibromas); and the remaining were SCCs. Among 84 tumors, HFUS correctly identified tumor margins in 81 cases, in agreement with histological findings. These results suggest that *ex vivo* ultrasound as a perioperative tool could increase surgeons’ confidence in accurately identifying surgical margins.^28^ In our cohort, we had significantly larger tumors which we could characterize thoroughly by HFUS without size limitation.

In another study on 65 patients with BCCs located on the face, scalp, trunk/extremities undergoing surgical removal with 2–3 mm surgical margins, *ex vivo* ultrasound (10–18 MHz) showed a specificity of 91.6% in correctly detecting tumor margins.^29^

**FIGURE 7:**
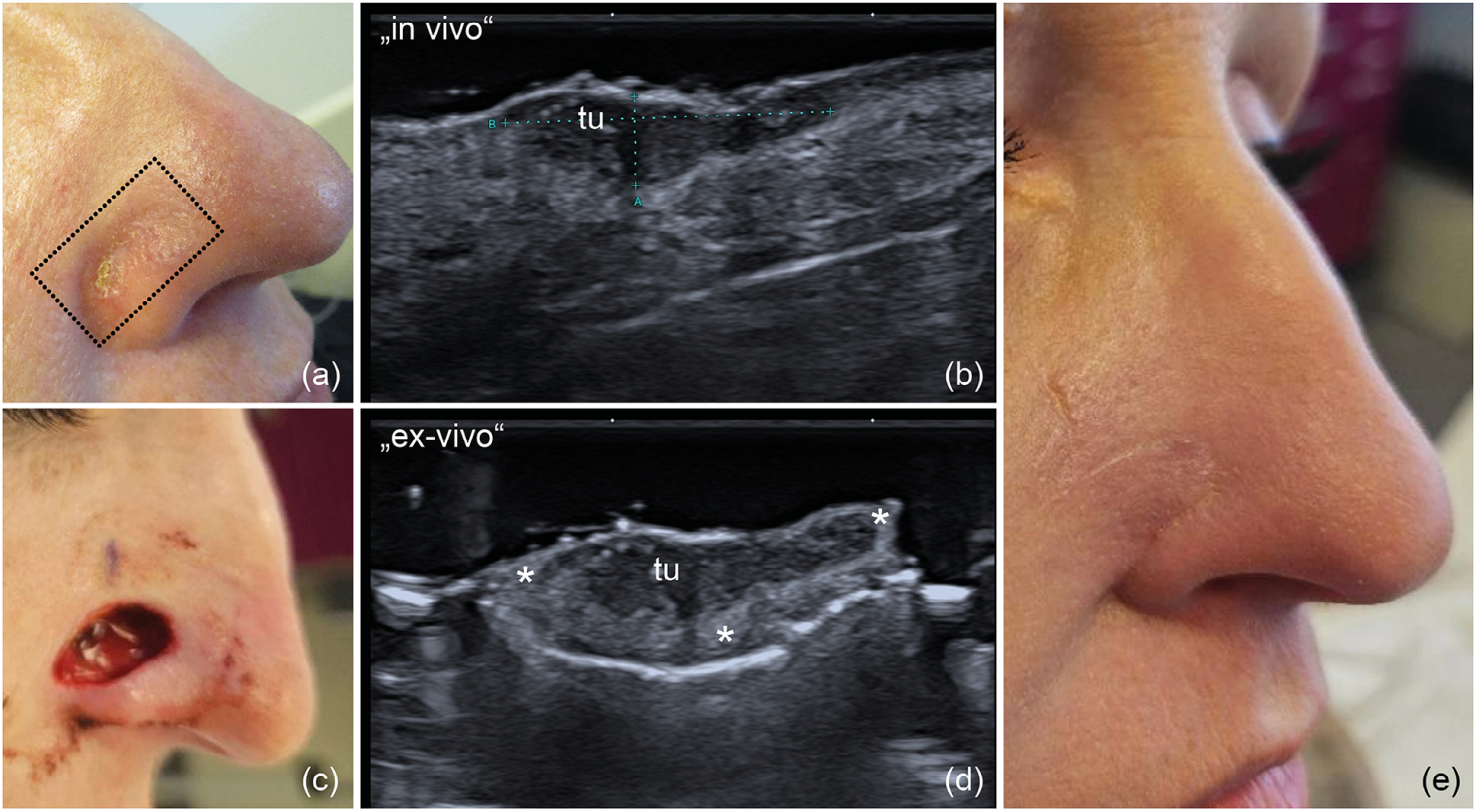
(a) Clinical aspect of an infiltrative BCC at the right nasal ala; the rectangle indicates the placement of the sonographic probe; (b) In vivo sonography displaying a hypoechoic lesion with hyperechoic spots, located in the dermis and extending into the subcutaneous tissue; (c) *ex vivo* sonography of the resected tumor, showing safety margins in depth and on both sides, marked by “*”; (d) clinical aspect of the surgical defect after tumor resection; (e) surgical site four weeks after defect reconstruction with a rhombic flap.

*Ex vivo* tumor margins assessment in our study showed a specificity of 98% or more both for SCC and BCC, with only five false‐negative and two false‐positive results, while the sensitivity was 60% and 66% for BCC and SCC, respectively. We correctly identified tumor‐free margins in 89% of the investigated lesions after the first resection, based on preoperative sonographic marking and *ex vivo* sonographic margin assessment. Additionally, eight R1 cases (6%) were detected, where re‐excision confirmed the presence of residual tumor (Figure 7). Overall, in our cohort, ultrasound demonstrated very high accuracy in detecting both tumor‐free margins and residual tumor (Table 2). In four BCC cases where *ex vivo* ultrasound indicated complete resection but histology revealed an R1 situation, one case was a basosquamous carcinoma with perineural infiltration, which was indeed missed by ultrasound. In two additional cases of helical sclerodermiform BCCs, histology revealed incomplete deep resection; however, partial excision of the underlying cartilage showed no tumor infiltration. In both cases, preoperative HFUS demonstrated an intact cartilage line beneath the lesion, which is why the resection was performed up to the cartilage. It is likely that the cartilage served as a barrier to tumor progression, as BCCs often spread along the cartilage before infiltrating it. In such cases, preoperative sonography may provide valuable information that, when supported by histological data, could help avoid extensive cartilage resections, complex reconstructions, and, in some instances, the need for cartilage grafting. One sclerodermiform BCC located in the parietal region was considered R1 on histology and re‐excised; however, no residual tumor was found. This might be explained by the fact that the histological assessment is done on a section of the tumor previously cut and prepared. In some cases, depending on the specimen sectioning, the histological assessment might also be false positive.

For one poorly differentiated SCC on the nose, which was falsely classified as R0 by ultrasound, histology revealed an R1 situation, and the patient required two additional re‐excisions. This was probably due to the fact that the tumor was very superficial laterally and situated in a highly photoexposed area, with abundant actinic elastosis, which is sometimes difficult to differentiate from tumor rest on ultrasound, as it also appears as a hypoechoic band of the dermis.^30^ For the moderately differentiated SCC in the parietal area, where ex vivo sonography indicated a potential deep R1 situation, re‐excision revealed no residual tumor. Therefore, the re‐intervention could have been avoided. High‐risk SCCs are usually irregularly shaped, have mixed echogenicity, and usually present perilesional inflammatory infiltrate, which can sometimes make it difficult to assess the borders correctly. Furthermore, when keratin on top of SCCs is not removed, the deep tumor border evaluation might be difficult to determine accurately, due to sonographic artifacts.

The use of in vivo and *ex vivo* ultrasound reduced the number of MCS stages by at least one in eight patients, who underwent re‐resection in a single surgical step. This resulted in a total reduction of eight hospitalization days. When considering the 121 primary R0 resections, in which *ex vivo* sonography indicated tumor‐free margins and this was confirmed histologically, there was a potential to reduce the total number of additional hospitalization days by at least 129. Considering the exponential increase in NMSC lately, these additional days could have been used for other patients, reducing overall waiting times and making healthcare resources more efficient.^31^ On the other hand, ultrasound recommended re‐excision in two R0 cases, which could have resulted in larger surgical defects requiring more complex reconstructions.

Mohs surgery, at least in the United States, is very expensive; in 2017, Medicare expenditures for Mohs surgery totaled $537 million, a significant increase compared to 2014. As the incidence of NMSC continues to rise, the demand for Mohs surgery will persist. Therefore, it is essential to develop strategies that not only reduce procedural costs but also minimize the number of surgical stages. Data suggest that even a 10% reduction in surgical stages per patient could save approximately $36 million annually.^18^, ^32^

In our analysis, we included only tumors of the head and face area. Out of 136 lesions, 38 BCCs and 9 SCCs were classified as high‐risk tumors based on their histological subtype. The number would be even higher when considering localization in the H‐zone, where MCS is the therapy of choice and the potential to reduce the number of surgeries remains an unmet need. We showed that even in infiltrative, sclerosing BCCs and low differentiated SCCs, *ex vivo* HFUS can correctly identify tumor margins and establish the need for an immediate re‐resection if needed. For OCT, *ex vivo* margin assessment is most accurate in nodular BCCs, compared to infiltrative/morpheaform tumors, where often a disagreement between histology and OCT was seen.^18^, ^33^ Confocal microscopy can also be used to assess tumor margins *ex vivo*; however, the tissue must be fixed, stained, scanned, and evaluated by a pathologist, which requires more time and resources compared to ultrasound.^10^

High‐frequency ultrasound is, on the other hand, widely available, enabling a very fast assessment of tumor margins both in vivo and *ex vivo*. It remains the only imaging technique which can define tumor depth without any penetration issues, characterize the primary tumor in all axes and perform a locoregional staging when indicated, and at ≥ 15 MHz it was shown to have a higher spatial resolution than PET‐CT and MRI.^34^

**FIGURE 8:**
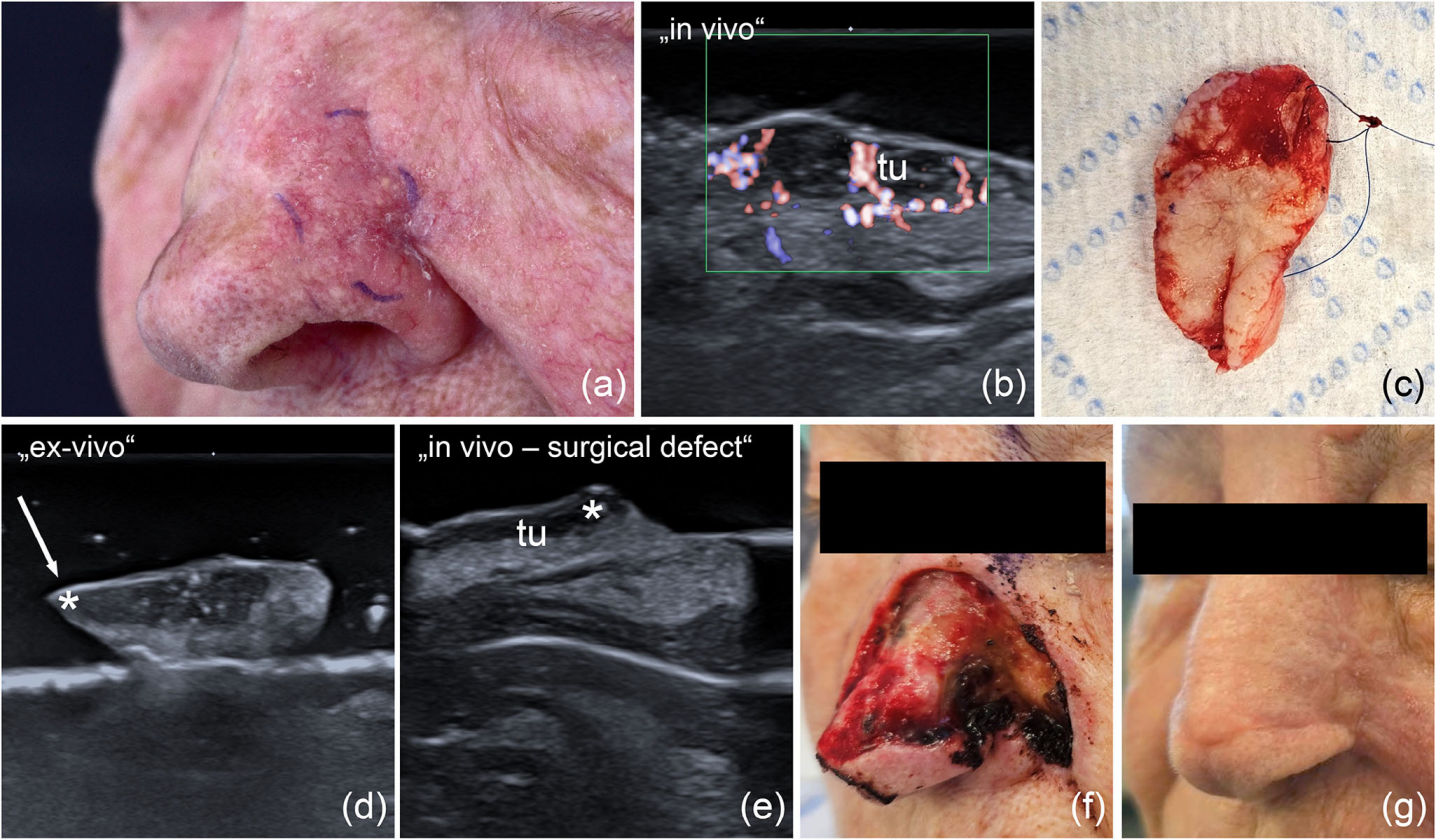
(a) Clinical aspect of a sclerodermiform BCC at the left nasal ala; (b) In vivo sonography displaying a hypoechoic lesion with hyperechoic spots and increased vascularization, located in the dermis and subcutaneous tissue, marked by “tu”; (c) surgical specimen after first excision, prepared for *ex vivo* sonography; (d) *ex vivo* sonography of the resected tumor, showing one side still infiltrated by the tumor, marked by “*”; (e) In vivo sonography of the surgical defect, showing residual tumor at one surgical margin, marked by “*”; (f) clinical aspect of the surgical defect after complete tumor resection; (g) surgical site 6 weeks after defect reconstruction with an advancement flap from the glabella and nasolabial fold.

*Ex vivo* HFUS can be performed on the freshly resected specimen prior to histopathological evaluation, serving as a bedside procedure for determining tumor margins and enabling immediate therapeutic decision‐making. This approach could spare patients additional surgical interventions and the periprocedural pain associated with infiltration anesthesia, allowing for faster defect closure. Moreover, in cases where *ex vivo* sonography clearly indicates tumor‐free margins, the need to await histological assessment could potentially be eliminated, enabling immediate reconstruction of the surgical defect. Another potential application during MCS is performing in vivo ultrasound of the surgical defect in cases of R1 margins and measuring the size of any residual tumor. This could enable a localized, tissue‐sparing excision, particularly in highly esthetic areas (Figure 8).

One limitation of HFUS is the difficulty in accurately identifying superficial lateral tumor margins in photo‐exposed areas due to the subepidermal low echogenicity band (SLEB), a hypoechoic area in the upper dermis resulting from chronic sun damage. Additionally, superficial perineural infiltration, such as single cells or small nests – as seen in our case – may be missed on ultrasound. In such situations, other imaging modalities with higher resolution of the upper skin layers, such as CM or LC‐OCT, should be considered.^35^ Another option is to complement the HFUS examination with ultra‐high frequency ultrasound (UHFUS) devices, currently operating at 50–70 MHz, which provide improved detection of superficial tumors with an axial spatial resolution of up to 30 µm.^36^ Ultra‐high frequency ultrasound has been successfully used in BCCs, showing an interclass correlation between ultrasound and histology of ≥ 0.8, and has also been applied in superficial SCCs.^5^, ^37^, ^38^ However, these devices are available in only a few centers worldwide, whereas HFUS is widely accessible.

Nonetheless, in rare uncertain cases – particularly with high‐risk facial tumors that often require local flaps for reconstruction – patients should be informed that *ex vivo* ultrasound may falsely identify tumor‐free margins (for example, in cases of perineural infiltration or superficial tumor nests in photo‐exposed areas). Therefore, histological confirmation should be awaited before defect closure. In cases of primary closure, a local re‐excision is usually not problematic. However, re‐resection after flap reconstruction can be challenging, as it may complicate the identification of residual tumor. For cases in which *ex vivo* ultrasound clearly identifies tumor margins, closure of the defect can be discussed with the patient beforehand. Nevertheless, it is important to note that HFUS requires well‐trained dermatologists, as it is an operator‐dependent examination that may produce certain artifacts, potentially leading to misinterpretation of sonographic findings.^39^

## CONCLUSIONS

The need for a method to identify tumor margins immediately during surgery, comparable to histology, has driven the development and investigation of various imaging techniques. Based on the present study, HFUS has the potential to accurately identify tumor margins both in vivo and *ex vivo*. Its main advantages include widespread availability, rapid perioperative application, time savings, and the provision of essential information to surgeons for an optimized surgical procedure. Furthermore, the routine use of HFUS in the dermatosurgical setting – especially during Mohs surgery or micrographic‐controlled surgery for NMSCs –could significantly reduce the number of surgeries and hospitalization time for patients, thereby lowering associated costs (operating rooms, personnel, pathology services). However, this approach should be validated in larger, prospective multicenter studies. In unclear cases involving high‐risk tumors with perineural infiltration or very superficial sclerodermiform lesions – particularly in sun‐exposed areas – a final histological assessment should still be awaited prior to defect closure.

## CONFLICT OF INTEREST STATEMENT

None.
